# Acute Effect of Evolocumab on Lipoprotein(a) Level and Inflammation in Patients with Coronary Artery Disease

**DOI:** 10.3390/jcdd9040101

**Published:** 2022-03-27

**Authors:** Seung Woo Choi, Joan Kim, Gyeong Won Jang, Young Shin Lee, Jin Sun Park, Jung Myung Lee, Hyung Oh Kim, Hyemoon Chung, Jong Shin Woo, Woo Shik Kim, Weon Kim

**Affiliations:** 1 Division of Cardiology, Department of Internal Medicine, Kyung Hee University Hospital, Kyung Hee University, Seoul 02447, Korea; swseungwoo@naver.com (S.W.C.); joankim6552@hanmail.net (J.K.); sculptor1232@naver.com (G.W.J.); conan2010@naver.com (Y.S.L.); cardioljm@gmail.com (J.M.L.); hypnotica1999@hanmail.net (H.O.K.); 111212@hanmail.net (H.C.); snowball77@hanmail.net (J.S.W.); wskim1125@khu.ac.kr (W.S.K.); 2 Department of Medicine, Graduate School of Medicine, Kyung Hee University, Seoul 02447, Korea; qkrwlstjs87@naver.com

**Keywords:** coronary artery disease, evolocumab, high-sensitivity C-reactive protein, lipoprotein(a), low-density lipoprotein cholesterol

## Abstract

Background: Several studies have shown that high plasma lipoprotein(a) concentrations are associated with an increased risk of arteriosclerotic cardiovascular disease. Thus, Lp(a) has emerged as a new therapeutic target. Circulating proprotein convertase subtilisin/kexin type 9 (PCSK9) inhibitors are new lipid-lowering agents that reduce low-density lipoprotein cholesterol as well as Lp(a). Methods: We analyzed the short-term effects of one-time administration of evolocumab (a PCSK9 inhibitor) on the lipid profiles (especially Lp(a)) and inflammatory markers in Korean patients with coronary artery disease (CAD) who underwent percutaneous coronary intervention (PCI). Sixty-four patients with CAD who underwent PCI were enrolled in this trial. Evolocumab (140 mg) was administered to patients within 24 h after PCI. Lipid profiles and inflammatory marker levels were measured at baseline and 2 weeks later. Results: The PCSK9 inhibitor significantly reduced the baseline levels of Lp(a) (−9.2 mg/dL, *p* < 0.001), but high-sensitivity C-reactive protein (+0.07 mg/dL, *p* = 0.272) was not significantly different after 2 weeks. In patients with an Lp(a) level of 50 mg/dL or more, the Lp(a) level decreased significantly by approximately 30%, from 95.6 mg/dL to 67.0 mg/dL (*p* < 0.001). Conclusions: One-time PCSK9 inhibitor treatment may be effective in lowering Lp(a) levels in Korean patients in the short term.

## 1. Introduction

In recent decades, most clinicians have focused their attention on the relationship between low-density lipoprotein cholesterol (LDL-C) and cardiovascular risk. However, despite having low LDL-C levels, some patients continue to be at a high cardiovascular risk. In this situation, lipoprotein(a) (Lp(a)) has become a new therapeutic target.

Although similar to LDL, Lp(a) is a lipoprotein that is produced by the covalent bonding of apolipoprotein(a) (apo(a)) to apolipoprotein B-100 (apoB) [1]. Several studies have shown that high plasma Lp(a) concentrations are associated with an increased risk of arteriosclerotic cardiovascular disease [2,3], presumably owing to the pro-atherogenic and pro-inflammatory effects of Lp(a). Based on recent studies on Lp(a), the 2019 European guidelines on dyslipidemia recommend measuring Lp(a) at least once in a lifetime [4].

Statins, which are widely used as the first-line treatment for dyslipidemia, have been reported to not affect Lp(a); rather, an increase of approximately 10% has been observed [5]. Circulating proprotein convertase subtilisin/kexin type 9 (PCSK9) inhibitors are new lipid-lowering agents that reduce LDL-C levels as well as those of Lp(a) [6].

Because most of the data on PCSK9 inhibitors are based on Western patients, this study aimed to evaluate the effect of PCSK9 inhibitors on Koreans. We analyzed the short-term effects of one-time administration of evolocumab (PCSK9 inhibitor) on the lipid profiles (especially Lp(a)) and inflammatory markers in Korean patients with coronary artery disease (CAD) who underwent percutaneous coronary intervention (PCI).

## 2. Materials and Methods

This was a retrospective, observational study. Sixty-four patients with CAD who underwent PCI were enrolled between March 2020 and May 2020. Patients with CAD included those with chronic stable angina and unstable angina. Patients with an active infection, severe liver failure, severe renal failure (creatinine clearance < 30 mg/dL), and myocardial infarction (MI) were excluded.

Evolocumab (140 mg) was subcutaneously injected within 24 h after PCI. Total cholesterol, triglyceride, high-density lipoprotein cholesterol, LDL-C, apo(a), apoB, phospholipid, Lp(a), and high-sensitivity C-reactive protein (hs-CRP) levels were measured before evolocumab administration. Outpatient follow-up was performed approximately 2 weeks after discharge (median days: 14.1 ± 7.3), and the blood tests performed during hospitalization were re-evaluated. All blood tests were performed in the fasting state. We compared the lipid profiles and inflammatory markers between baseline and 2 weeks later.

LDL-C was analyzed by a homogeneous assay. Lp(a) was analyzed by nephelometry, and 30 mg/dL or less was determined as a normal level. Hs-CRP was analyzed by a turbidimetric immunoassay, and 0.1 mg/dL or less was determined as a normal level.

To analyze serial changes in blood tests, paired t-tests were performed. We used SPSS for data analysis (version 17.0; IBM Corp., Armonk, NY, USA). Subgroup analysis was performed based on an Lp(a) level of 50 mg/dL. This was based on some studies that showed a significant association between Lp(a) reduction and major adverse cardiovascular events (MACE) at the Lp(a) level of 50 mg/dL or higher [7]. In the subgroup analysis of hs-CRP, its cut-off value was set to 0.3 mg/dL. Statistical significance was set at *p* < 0.05. This study was approved by the institutional review board of Kyunghee University Hospital.

## 3. Results

Among the 64 patients, 40 (62%) were male; the average age was 68.3 ± 9.7 years. Additionally, 33% had diabetes mellitus, 10% were current smokers, 80% had hypertension, and 81% were on statins for hyperlipidemia. Additional details of the patients are presented in Table 1.

The changes in blood tests before and 2 weeks after evolocumab administration are shown in Table 2. The levels of total cholesterol, triglyceride, LDL-C, apoB, phospholipid, and Lp(a) were significantly decreased after 2 weeks. The LDL-C level decreased by approximately 58%, from 89.5 mg/dL to 37.2 mg/dL. The non-HDL-C level also decreased by approximately 64% from 104.4 mg/dL to 37.8 mg/dL. The levels of apoB and phospholipid were decreased by approximately 41% and 22%, respectively. The Lp(a) level decreased by approximately 27%, from 33.5 mg/dL to 24.4 mg/dL. However, the levels of high-density lipoprotein cholesterol, apoA1, and hs-CRP were not significantly different, regardless of evolocumab administration.

**Figure 1:**
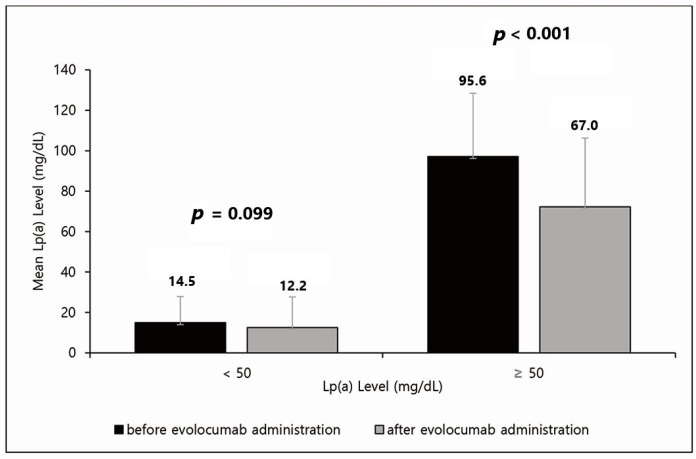
Effect of evolocumab based on an Lp(a) level of 50 mg/dL. In patients with Lp(a) < 50 mg/dL, Lp(a) levels decreased by approximately 16%, from 14.5 mg/dL to 12.2 mg/dL, but this difference was not significant (*p* = 0.099). In patients with Lp(a) ≥ 50 mg/dL, Lp(a) levels decreased significantly by approximately 30%, from 95.6 mg/dL to 67.0 mg/dL (*p* < 0.001). Lp(a), lipoprotein(a).

The effect of the PCSK9 inhibitor was analyzed by dividing the patients into two groups based on an Lp(a) level of 50 mg/dL (Figure 1). Forty-nine patients had Lp(a) levels < 50 mg/dL and fifteen had Lp(a) levels ≥ 50 mg/dL. In patients with Lp(a) levels < 50 mg/dL, the Lp(a) level decreased by approximately 16%, from 14.5 mg/dL to 12.2 mg/dL, but this difference was not significant (*p* = 0.099). In patients with Lp(a) levels ≥ 50 mg/dL, the Lp(a) level decreased significantly by approximately 30%, from 95.6 mg/dL to 67.0 mg/dL (*p* < 0.001). Moreover, Lp(a) was not normally distributed, so we calculated the median values and IQR. The quartile values of pre-Lp(a) were 4.87, 17.15, and 47.40 in order. Additionally, the quartile values of post-Lp(a) were 3.11, 8.98, and 34.28 in order.

The effect of the PCSK9 inhibitor was next analyzed by dividing the patients into two groups based on an hs-CRP level of 0.3 mg/dL. Fifty-one patients had hs-CRP levels < 0.3 mg/dL and thirteen had hs-CRP levels ≥ 0.3 mg/dL. In patients with hs-CRP levels < 0.3 mg/dL, the hs-CRP level increased from 0.08 mg/dL to 0.18 mg/dL, but this difference was not significant (*p* = 0.102). In patients with hs-CRP levels ≥0.3 mg/dL, the hs-CRP level mildly increased from 0.79 mg/dL to 0.73 mg/dL, but this difference was not significant (*p* = 0.788).

## 4. Discussion

PCSK9 inhibitors are promising options for lipid-lowering therapy. The lipid-lowering effect of PCSK9 inhibitors is faster and more effective than that of statins [8]. In addition, unlike statins, PCSK9 inhibitors have an effect on Lp(a) reduction [9]. In our study, the lipid-lowering effect of this PCSK9 inhibitor was exerted quickly and effectively in Korean patients.

Lp(a) is an independent risk factor for cardiovascular disease [10]. Although the evidence that lowering Lp(a) lowers the risk of cardiovascular disease is still insufficient, some studies suggest that significant modulation of Lp(a) may have cardiovascular benefits. According to the results of a Mendelian randomization analysis from five studies, significant absolute reductions in Lp(a) of about 100 mg/dL may be required to produce a similar preventive effect of cardiovascular disease as to what can be achieved by lowering the LDL-C level by 39 mg/dL [11]. In our study, Lp(a) levels were significantly and quickly reduced even with one-time administration of the PCSK9 inhibitor, especially in patients with Lp(a) levels ≥ 50 mg/dL. Generally, in about 1/3 of patients, evolocumab does not exert an Lp(a)-reducing effect. In patients with Lp(a) less than 50 mg/dl, there was hardly any Lp(a)-reducing effect, and in patients with Lp(a) above 50 mg/dl, it was confirmed that Lp(a) was reduced in all patients.

It is a well-known fact that the administration of PCSK9 inhibitors shows an improvement in lipid profiles including Lp(a). However, the data of PCSK9 inhibitors in Asians are limited, especially in Koreans. Through our trial, we confirmed that a PCSK9 inhibitor in Koreans showed a similar decrease in lipid profiles to that in other races. It is also interesting to note that not only the LDL cholesterol level, but also apoB and phospholipid levels were improved. Furthermore, since our study confirmed the short-term effect of a single injection of a PCSK9 inhibitor, we may conduct a follow-up study on the role of PCSK9 inhibitors as a pre-treatment before PCI.

hs-CRP is an inflammatory marker that reflects cardiovascular risk. Usually, statin-based treatment is known to decrease hs-CRP levels. PCSK9 inhibitors are known to not be related to decreases in hs-CRP levels. Therefore, we planned our trial to confirm if the short-term effect of a PCSK9 inhibitor could lower hs-CRP levels. Unfortunately, the results did not show any significant difference.

The limitations of our study are as follows. First, a small group of 64 patients with chronic stable angina and unstable angina pectoris (except MI) was enrolled. We tried to maintain as much homogeneity as possible by excluding MI patients, but nevertheless, 64 patients is a very small group. Second, it is impossible to derive a clinical outcome by monitoring the lipid-lowering effect for a short period of only 2 weeks. While short-term effects of the PCSK9 inhibitor were confirmed, information such as long-term effects of the PCSK9 inhibitor or changes in diet and lifestyle could not be confirmed. Third, the statistical power was weak because this is not a prospective randomized study and there was no control group. This is a significant limitation. Fourth, baseline statin use was not uniform.

However, clinical data on PCSK9 inhibitors are insufficient in East Asia. Although our study analyzed only the short-term effects of a PCSK9 inhibitor, we have shown that the effects were similar to those observed in existing studies outside East Asia. Additionally, in the short term, there was no significant decrease in hs-CRP levels, but significant decreases were observed in Lp(a) levels.

## 5. Conclusions

This study shows that a single administration of the PCSK9 inhibitor evolocumab significantly and rapidly reduced Lp(a) levels in the short term, especially when the Lp(a) level was above 50 mg/dL.

## Figures and Tables

**Table 1:** Clinical characteristics.

Variable	*n* = 64
Age (years)	68.3 ± 9.7
Male, *n* (%)	40 (62)
Diabetes mellitus, *n* (%)	21 (33)
Arterial hypertension, *n* (%)	51 (80)
Hyperlipidemia, *n* (%)	52 (81)
Statin monotherapy	29 (56)
Statin + ezetimibe combination	23 (44)
Active smoker, *n* (%)	10 (16)
Previous PCI, *n* (%)	25 (39)
Diagnosis, *n* (%)	
Chronic stable angina	51 (80)
Unstable angina	13 (20)
Lipid profile and inflammatory marker	
Total cholesterol (mg/dL)	154.2
Triglyceride (mg/dL)	139.1
HDL cholesterol (mg/dL)	49.8
LDL cholesterol (mg/dL)	89.5
Lp(a) (mg/dL)	33.5
Hs-CRP (mg/dL)	0.22

Abbreviations: PCI, percutaneous coronary intervention.

**Table 2:** Changes in blood tests from the baseline to 2 weeks after evolocumab administration.

Parameter	Week 0 Mean (SD)	Week 2 Mean (SD)	Change Mean	*p*
Total-C (mg/dL)	154.2 (40.2)	86.7 (21.3)	−67.5	0.000
TG (mg/dL)	139.1 (84.6)	107.0 (80.8)	−32.0	0.002
HDL-C (mg/dL)	49.8 (12.7)	48.9 (11.9)	−0.84	0.369
LDL-C (mg/dL)	89.5 (29.3)	37.2 (14.3)	−52.3	0.000
Non-HDL-C (mg/dL)	104.4 (36.8)	37.8 (16.9)	−66.6	0.000
Apo(a) (mg/dL)	126.3 (22.6)	123.5 (20.6)	−2.8	0.159
apoB (mg/dL)	70.6 (19.8)	41.9 (4.0)	−28.9	0.000
phosphoL (mg/dL)	185.1 (36.7)	144.2 (27.1)	−41.0	0.000
hs-CRP (mg/dL)	0.22 (0.52)	0.29 (0.62)	+0.07	0.272
Lp(a) (mg/dL)	33.5 (39.1)	24.4 (30.1)	−9.2	0.000

Abbreviations: apo(a), apolipoprotein(a); apoB, apolipoprotein B; HDL-C, high-density lipoprotein cholesterol; hs-CRP, high-sensitivity C-reactive protein; LDL-C, low-density lipoprotein cholesterol; Lp(a), lipoprotein(a); TG, triglyceride.

## Data Availability

They are available from the corresponding authors upon reasonable request.
